# Responses of stable vegetation to drought accumulation timescales and time lags in the Weihe River Basin

**DOI:** 10.3389/fpls.2026.1974112

**Published:** 2026-09-30

**Authors:** Xiangxiang Hu, Yaya Shi

**Affiliations:** 1 School of Resources and Environmental Engineering, Tianshui Normal University, Tianshui, Gansu, China; 2 Research Center for Disaster Early Warning and Intelligent Prevention and Control in the Weihe River Basin, Tianshui, Gansu, China

**Keywords:** drought accumulation timescale, enhanced vegetation index, stable vegetation, standardized precipitation evapotranspiration index, Weihe River Basin, window-end lag

## Abstract

**Introduction:**

This study aimed to distinguish drought accumulation timescale from response timing in stable vegetation in the Weihe River Basin.

**Methods:**

We analyzed MODIS enhanced vegetation index (EVI) and ERA5-Drought standardized precipitation evapotranspiration index (SPEI) data for 2003–2024 on the native 0.25° drought grid. Persistent land-cover classification required at least 18 valid annual classifications and a modal-class fraction of at least 80%. Four accumulation timescales (1, 3, 6, and 12 months) and 13 window-end lags (0–12 months) formed 52 response–exposure combinations. We controlled for growing-season month effects and a linear trend. We applied maximum-statistic tests with synchronized 2-year moving-block resampling and controlled the false discovery rate across cells using the Benjamini–Hochberg procedure.

**Results:**

The area-weighted mean of grid-cell partial correlations peaked at SPEI-12, lag 0 (r = 0.3216), with a mean slope of 0.00971 EVI units per SPEI unit. Of 190 eligible cells, 126 showed a corrected positive association, covering 69.68% of eligible stable-vegetation area. Grasslands and high-elevation areas had stronger descriptive responses. Relaxing valid-area coverage from 0.70 to 0.50 retained the basin-wide peak. Across detected cells, the median bootstrap reselection frequency of the observed best timescale–lag combination was 53.1%. Across 2,252 SPEI-3 grid-cell drought events, the mean EVI residual reached −0.00909 one month after onset (95% onset-year block-bootstrap interval, −0.01448 to −0.00439). Among 2,105 uncensored events, meteorological drought termination was confirmed a median of 3 months after the last dry month (interquartile range, 2–3 months).

**Discussion:**

The dominant association links vegetation condition to the 12-month water balance ending in the response month. These findings support monitoring that combines long-term water balance, early vegetation responses, and continued vegetation observations after meteorological drought termination.

## 1 Introduction

Drought develops when water supply remains below ecosystem demand, with impacts shaped by its duration, severity, and spatial extent (Wilhite and Glantz, 1985). Warming can intensify evaporative demand and alter terrestrial water balance, increasing drought risk and ecological impacts in some regions (Dai, 2013; Trenberth et al., 2014). Severe drought can suppress photosynthesis and productivity, weaken carbon uptake, and cause tree mortality (Reichstein et al., 2013; Allen et al., 2010; Xu et al., 2019). Recent rainfall-manipulation experiments also show that grassland productivity losses can accumulate during consecutive drought years, with substantial differences between continents (Yu et al., 2025). Drought assessment therefore needs to consider ecosystem sensitivity, persistent effects, and meteorological deficits (Crausbay et al., 2017; Ault, 2020). In the Weihe River Basin, vegetation restoration, agricultural production, and urban water use compete for limited water resources. Evidence from the surrounding Loess Plateau further shows that vegetation greening need not imply increasing resilience (Wang et al., 2023b). Identifying when established vegetation responds to water deficits can thus inform drought-risk screening beyond trends in greenness alone.

Long satellite records and multiscalar drought indices provide an observational basis for regional vegetation–drought studies. The enhanced vegetation index (EVI) reduces soil-background and some atmospheric effects while retaining sensitivity to relatively dense canopies (Huete et al., 2002). The standardized precipitation evapotranspiration index (SPEI) combines precipitation and potential evapotranspiration over specified accumulation windows (Beguería et al., 2014). Characteristic response timescales vary among biomes and climatic settings (Ji and Peters, 2003; Vicente-Serrano et al., 2013; Gouveia et al., 2017). Global analyses likewise identify spatially heterogeneous climate sensitivity, effects of antecedent precipitation, and disproportionate productivity losses during extreme events (Seddon et al., 2016; Papagiannopoulou et al., 2017; Zscheischler et al., 2014). Recent studies explicitly examine cumulative and lagged responses across China and the Yellow River Basin (Zhan et al., 2022; Wang et al., 2023a). Other work shows that hydroclimatic context affects steppe phenology and that responses to compound drought–heat stress vary by season (Liu et al., 2024; Li et al., 2026). These findings emphasize the importance of defining the exposure window, response variable, and seasonal context when comparing response timing.

Accumulation timescale and window-end lag describe different temporal dimensions. The accumulation timescale specifies how many months of water balance contribute to a drought index. The window-end lag is the interval between the end of that accumulation window and the vegetation observation. Longer accumulation windows can reflect water availability over the preceding months. Antecedent conditions can affect subsequent vegetation state, and drought legacies can extend across seasons (Papagiannopoulou et al., 2017; Bastos et al., 2020; Wu et al., 2022). Phenology can also alter ecological recovery after drought (Li et al., 2023). Land-cover classification uncertainty and interannual conversion introduce additional difficulties when comparing vegetation types (Friedl et al., 2010). Joint analysis therefore requires distinct definitions of accumulated exposure, response timing, and drought recovery.

Existing studies establish the importance of cumulative and lagged effects, but several issues remain when interpreting them jointly at the basin scale. First, reported optima depend on the response variable, candidate windows, and the position of each exposure window relative to vegetation observations. A shared two-dimensional timescale–lag definition is therefore needed for comparable estimates. Second, selecting a maximum from many correlated combinations creates uncertainty beyond that of a single correlation. Temporal dependence, spatial correlation, and repeated testing must be considered together. Block resampling provides a basis for inference with dependent observations, while false-discovery-rate control addresses multiple spatial tests (Künsch, 1989; Benjamini and Hochberg, 1995). Third, spatial resolution affects ecological patterns, and spatial dependence complicates their statistical interpretation (Wu, 2004; Dormann et al., 2007). Resampling coarse drought data onto fine vegetation pixels does not create independent drought observations. Quality filtering, valid-area coverage, and persistent land-cover classification are also needed to limit confounding by missingness and land conversion. A basin-scale framework should therefore connect explicitly defined exposure windows, selection-aware inference, and event trajectories.

We investigated vegetation with persistent land-cover classification in the Weihe River Basin during 2003–2024 using the native 0.25° ERA5-Drought grid. We addressed three questions: (1) which accumulation-timescale and window-end-lag combination has the strongest basin-wide EVI association, and where are positive associations detectable; (2) how do response surfaces differ among vegetation types and environmental settings; and (3) how do EVI anomalies evolve around drought onset, and when is meteorological drought termination confirmed? Quality-controlled monthly EVI and annual land cover defined the response population. Four SPEI accumulation timescales and 13 window-end lags formed 52 combinations evaluated with dependence-aware inference. Alternative vegetation products were screened under the same eligibility rules, and event composites described seasonal drought trajectories. By separating accumulated exposure, window-end lag, and meteorological termination, the study provides temporally explicit evidence for monitoring established vegetation in a semi-humid to semi-arid transition basin.

## 2 Study area and data

### 2.1 Study area

The Weihe River Basin lies in the central Yellow River Basin from 104.03° to 110.25°E and from 33.72° to 37.40°N and covers approximately 134,200 km². The basin spans parts of Gansu and Shaanxi provinces and the Ningxia Hui Autonomous Region. Elevation generally decreases from west to east, and the basin contains plateau mountains upstream, loess hills in the middle reaches, and valley plains downstream. The mainstem Weihe River and its associated urban–agricultural corridor extend predominantly from west to east (**Figure 1**). Strong relief and longitudinal hydroclimatic gradients create pronounced spatial heterogeneity in vegetation composition and water availability.

**Figure 1:**
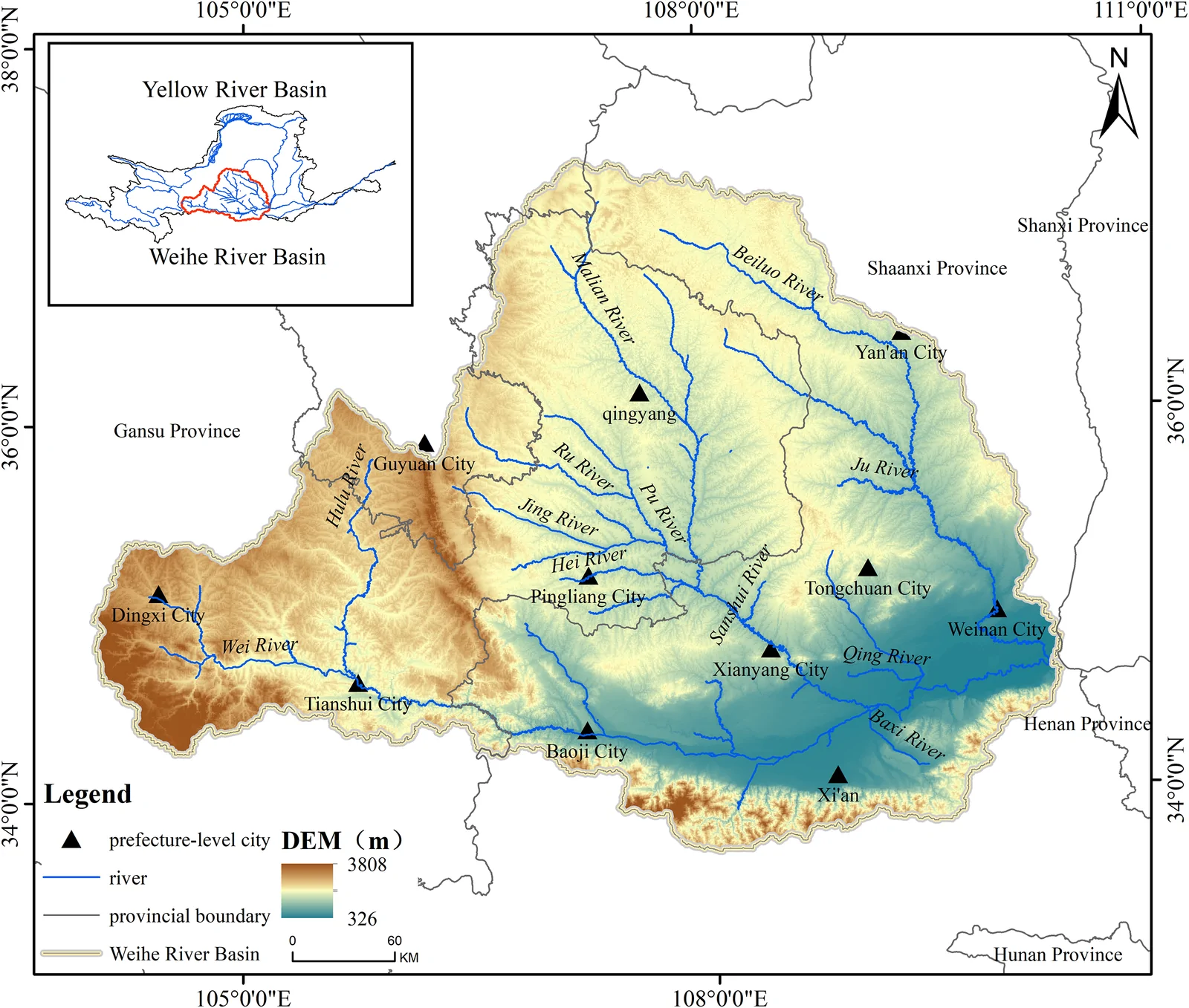
Geographic location and topographic setting of the Weihe River Basin. The topographic base layer was derived from Shuttle Radar Topography Mission (SRTM) elevation data at 3-arc-second (approximately 90 m) spacing (Farr et al., 2007). Blue lines show major rivers, gray lines show provincial boundaries, black triangles indicate prefecture-level cities, and the inset shows the Weihe River Basin within the Yellow River Basin together with their river networks.

Stable vegetation is dominated by forest, grassland, and cropland. Agricultural use is concentrated in low-elevation valleys and plains, whereas natural or restored vegetation is more prevalent in mountainous and plateau areas. Meteorological drought, soil-water availability, atmospheric demand, and irrigation management therefore operate differently across landform units, making the basin well suited to evaluating accumulation timescales, window-end lags, and spatially heterogeneous vegetation responses.

### 2.2 Data sources

The response-analysis period was January 2003–December 2024. ERA5-Drought SPEI from January 2002 onward supplied antecedent index values for lags of up to 12 months; each index already incorporated its stated accumulation window. The MODIS composite beginning in December 2002 was retained where it overlapped January 2003. The ERA5-Land and TerraClimate source records included data from 2002 onward. Environmental stratification used soil-moisture and VPD data for the 264 months of 2003–2024. **Table 1** distinguishes the analysis period from the earlier records used to define antecedent conditions. Quality flags were used to retain valid observations; missing values were not temporally interpolated. The basin boundary was taken from the archived WeiHeWaterShed.shp polygon. It was reprojected from WGS84 Lambert Conformal Conic to WGS84 geographic coordinates, dissolved, and repaired. Polygon fragments and interior holes smaller than 0.01 km² were removed during geometry cleaning.

**Table 1:** Study datasets, spatiotemporal resolutions, and uses.

Data product	Variable	Period and temporal resolution	Spatial resolution	Role and source
Weihe River Basin boundary	Basin polygon	Static	Vector	Spatial mask and grid intersections; archived WeiHeWaterShed.shp polygon, reprojected and geometry-cleaned (Section 2.2)
MOD13A1.061	EVI, VI_Quality, pixel reliability	2003–2024; 16-day; late-2002 overlap context	500 m	Primary response; Didan (2021); doi: 10.5067/MODIS/MOD13A1.061
ERA5-Drought v1.0	SPEI-1, SPEI-3, SPEI-6, SPEI-12	2002–2024; monthly	0.25°	Primary exposure and lag context; Copernicus Climate Change Service (2025); doi: 10.24381/9bea5e16
MCD12Q1.061	LC_Type1, QC	2003–2024; annual	500 m	Stable classes; Friedl and Sulla-Menashe (2022); doi: 10.5067/MODIS/MCD12Q1.061
Copernicus DEM GLO-30	Elevation	Static	1 arc-second (about 30 m)	Elevation strata; European Space Agency (2021); doi: 10.5270/ESA-c5d3d65
ERA5-Land	SWVL1–SWVL3	2003–2024; monthly	0.1°	Soil moisture; Muñoz-Sabater et al. (2021); data doi: 10.24381/cds.68d2bb30
TerraClimate v1.1	VPD	2003–2024; monthly	1/24°	Atmospheric-dryness strata; Abatzoglou et al. (2018)
FAO GMIA v5	Area equipped for irrigation	Approximately 2005; static	5 arc-min	Irrigation-background strata; Siebert et al. (2013)
MCD15A3H.061/ MOD17A2HGF.061	LAI and QC/GPP and QC	2003–2024; 4-day/8-day	500 m	Alternative responses; Myneni et al. (2021)/ Running and Zhao (2021); product DOIs in Section 2.2.2

All response analyses cover 2003–2024. Antecedent SPEI values from 2002 supply lag context; the accumulated water-balance history is already encoded in each SPEI index. A late-2002 MODIS composite contributes only where it overlaps January 2003. ERA5-Land and TerraClimate data used in the analyses cover January 2003–December 2024, although their source records also included 2002. All analyses use the native 0.25° ERA5-Drought grid, without resampling SPEI to 500 m.

#### 2.2.1 Primary analysis data

Vegetation response was represented by 16-day MOD13A1.061 EVI, VI_Quality, and pixel-reliability layers (Didan, 2021; doi: 10.5067/MODIS/MOD13A1.061). Monthly ERA5-Drought version 1.0 provided SPEI values, standardized against 1991–2020, for 1-, 3-, 6-, and 12-month accumulation periods (Copernicus Climate Change Service, 2025; doi: 10.24381/9bea5e16). Annual MCD12Q1.061 IGBP land cover and its quality layer identified persistent vegetation classes during 2003–2024 (Friedl and Sulla-Menashe, 2022; doi: 10.5067/MODIS/MCD12Q1.061).

#### 2.2.2 Auxiliary stratification and robustness data

Environmental stratification used Copernicus DEM GLO-30 elevation (European Space Agency, 2021; doi: 10.5270/ESA-c5d3d65) and monthly ERA5-Land soil moisture (Muñoz-Sabater et al., 2021; data doi: 10.24381/cds.68d2bb30). Volumetric water content at 0–7, 7–28, and 28–100 cm supplied a thickness-weighted 0–100 cm diagnostic. Additional covariates were monthly TerraClimate version 1.1 vapor pressure deficit (VPD; Abatzoglou et al., 2018) and the GMIA version 5 equipped-irrigation fraction for approximately 2005 (Siebert et al., 2013). These environmental variables supported descriptive stratification of vegetation responses across the basin. MCD15A3H.061 leaf area index (LAI; Myneni et al., 2021; doi: 10.5067/MODIS/MCD15A3H.061) and MOD17A2HGF.061 gross primary productivity (GPP; Running and Zhao, 2021; doi: 10.5067/MODIS/MOD17A2HGF.061) supplied alternative vegetation responses.

## 3 Methods

### 3.1 Analytical workflow

The workflow comprised three stages (**Figure 2**). First, MODIS EVI was quality controlled, composited monthly, and aggregated to the native 0.25° ERA5-Drought grid. Land-cover data identified stable vegetation, while terrain and environmental variables supported stratification. Second, vegetation responses were estimated across 52 combinations of four accumulation timescales and 13 window-end lags, accounting for temporal dependence and spatial multiple comparisons. Third, parallel analyses examined differences among vegetation types and environmental settings, along with drought-event trajectories and meteorological drought termination. Sensitivity checks evaluated sensitivity to coverage thresholds, the use of rank correlation, and SPEI extremes. We also assessed whether alternative vegetation responses met the eligibility criteria and how their response patterns compared with EVI.

**Figure 2:**
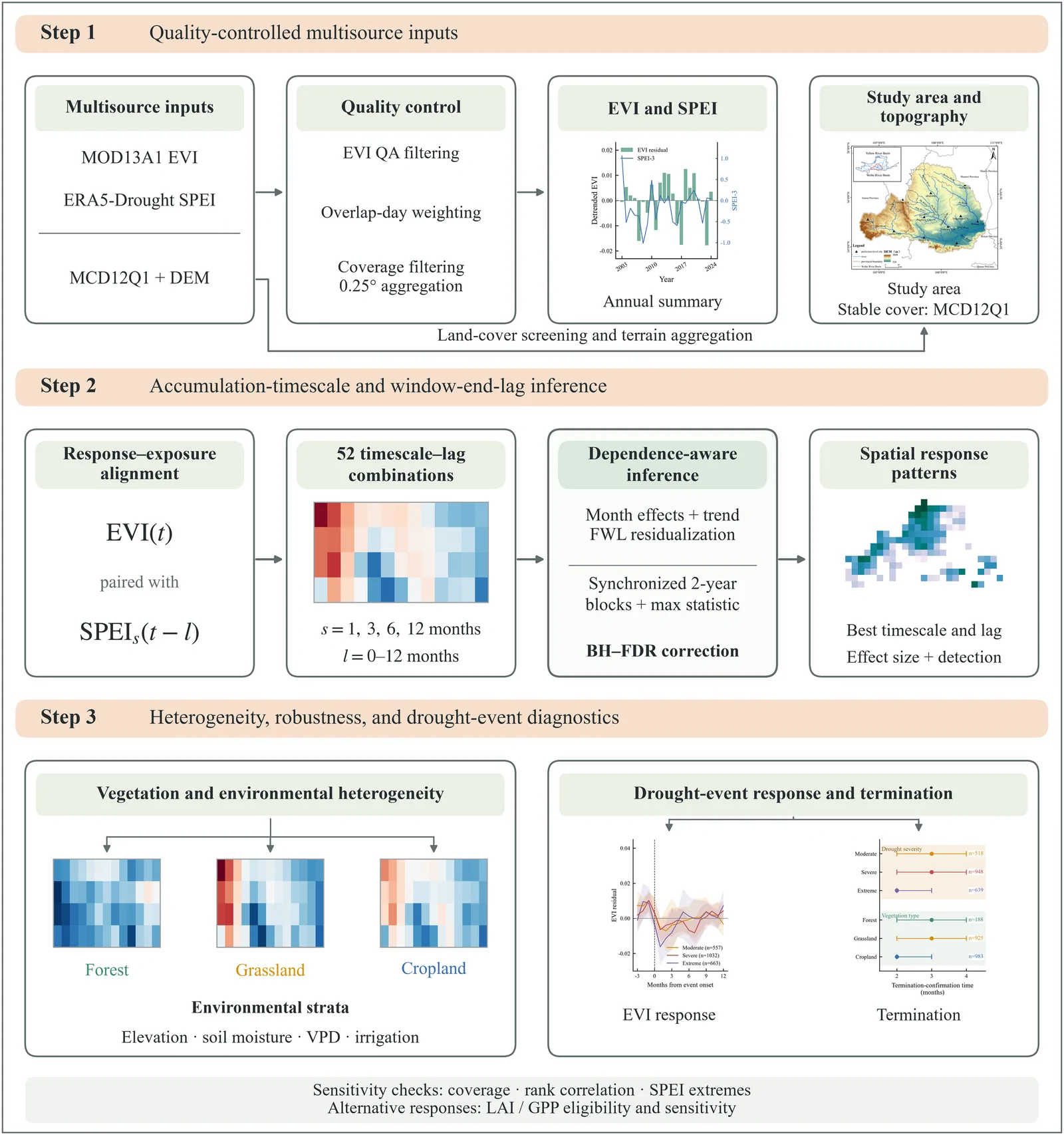
Data processing and analytical workflow. Step 1 comprises MODIS EVI quality control, monthly compositing, and aggregation to the native 0.25° ERA5-Drought grid. Land cover identifies stable vegetation, while terrain and environmental variables support stratification. Step 2 estimates vegetation responses across 52 timescale–lag combinations and accounts for temporal dependence and spatial multiple comparisons. Step 3 examines vegetation and environmental heterogeneity, robustness, sensitivity to alternative vegetation responses, drought-event trajectories, and meteorological drought termination. The annual EVI and SPEI summary is illustrative; the analyses use monthly data.

### 3.2 EVI quality control and monthly compositing

MOD13A1 EVI was scaled by 0.0001. The primary filter accepted MODLAND QA values no greater than 1 and officially acceptable VI-usefulness classes. It excluded high aerosol load, adjacent or mixed clouds, snow or ice, and shadow contamination, and required pixel-reliability values of 0 or 1. A strict sensitivity filter additionally required MODLAND QA and pixel reliability to equal 0. Each 16-day composite was weighted by the days overlapping a calendar month. For MODIS pixel *i*, calendar month m, and composite k, $K_{m}$ is the set of composites intersecting month m. $EVI_{i,k}$ is composite EVI, $w_{k,m}$ is the overlap in days, and $q_{i,k}$ equals 1 for an observation passing the quality filter and 0 otherwise. Monthly EVI, denoted $EVI_{i,m}^{mon}$, was calculated as

(1) $$\text{EV}I_{i,m}^{\text{mon}}=\frac{\sum_{k\in K_{m}}w_{k,m}q_{i,k}\text{EV}{\text{I}}_{i,k}}{\sum_{k\in K_{m}}w_{k,m}q_{i,k}}$$

Temporal coverage was defined as the valid overlap weight divided by the total possible overlap weight:

(2) $$C_{i,m}=\frac{\sum_{k\in K_{m}}w_{k,m}q_{i,k}}{\sum_{k\in K_{m}}w_{k,m}}$$

Only monthly values with $C_{i,m}\geq \;0.70$ were retained, where $C_{i,m}$ is the temporal-coverage fraction. Quality-driven missingness remained missing and was not interpolated. The denominator in Equation 2 includes the total potential overlap contribution, so coverage lies between 0 and 1. A zero valid-weight denominator in Equation 1 yields a missing monthly value, not zero EVI.

### 3.3 Stable vegetation identification and common-grid aggregation

For annual MCD12Q1 classifications during 2003–2024, quality classes 0 and 9 were accepted. Each pixel was required to have at least 18 valid years, with its modal IGBP class representing at least 80% of those years. Thus, stable denotes persistent land-cover classification, not unchanged physiology or an identical class in every year. Accepted vegetation classes were IGBP classes 1–12 and 14. Pixels classified as water, urban land, permanent snow or ice, or barren land were excluded, as were pixels failing the year-count or modal-class criteria. Here, $n_{i}$ denotes the number of valid years, $p_{i}$ the modal-class fraction, $c_{i}^{*}$ the modal class, and V the accepted class set. The indicator function I(·) equals 1 when all enclosed conditions hold and 0 otherwise. The stable-vegetation indicator $M_{i}$ was defined by Equation 3:

(3) $$M_{i}=\text{I}(n_{i}\geq 18,p_{i}\geq 0.80,c_{i}^{*}\in \text{V})$$

Target-cell boundaries were constructed from ERA5-Drought coordinates and densified before projection to the MODIS sinusoidal equal-area coordinate system. Exact intersections were calculated among 500 m pixels, target cells, and the basin boundary. For target grid cell g, $A_{i,g}$ is the within-basin overlap area with pixel i. The indicator $V_{i,m}$ equals 1 when monthly pixel EVI passes the temporal-coverage requirement and 0 otherwise; it is distinct from the class set V. Grid-cell EVI, $EVI_{g,m}$, and the valid stable-vegetation area fraction, $F_{g,m}$, were calculated using Equation 4:

(4) $$\text{EV}{\text{I}}_{g,m}=\frac{\sum_{i}A_{i,g}M_{i}V_{i,m}\text{EV}{\text{I}}_{i,m}^{\text{mon}}}{\sum_{i}A_{i,g}M_{i}V_{i,m}},F_{g,m}=\frac{\sum_{i}A_{i,g}M_{i}V_{i,m}}{\sum_{i}A_{i,g}M_{i}}$$

The primary analysis required $F_{g,m}\geq \;0.70$. Grid-months with zero valid stable-vegetation area were missing; cells with no stable vegetation had no defined response. A sensitivity analysis relaxed the spatial valid-area threshold to 0.50 while retaining the 0.70 temporal-coverage requirement and all other eligibility criteria. Every target cell intersecting the basin retained its basin fraction and native MODIS support. SPEI was retained on its native 0.25° grid without resampling to 500 m.

### 3.4 Accumulation-timescale and window-end-lag model

The main analysis used the April–October growing season. For grid cell g and response month t, the accumulation timescale s was 1, 3, 6, or 12 months. The window-end lag l ranged from 0 to 12 months, giving 52 combinations. The exposure $X_{g,t}^{\left(s,l\right)}$ was the SPEI value for timescale s in month t−l, defined as in Equation 5:

(5) $$X_{g,t}^{\left(s,l\right)}=\text{SPE}{\text{I}}_{g,t-l}^{\left(\text{s}\right)},s\in \{1,3,6,12\},l\in \{0,\ldots ,12\}$$

The associated water-balance window is [t−l−s+1,t−l], so the maximum look-back is 23 months. Lag 0 means that the accumulation window ends in the response month; it denotes a concurrent association rather than a causal delay. Each cell and combination used exactly matched valid response–exposure pairs in the following model:

(6) $$Y_{g,t}=\sum_{j=4}^{10}\delta_{g,j}D_{j,t}+\gamma_{g}\tau_{t}+\beta_{g,s,l}X_{g,t}^{\left(\text{s},\text{l}\right)}+\epsilon_{g,t}$$

Here, $Y_{g,t}$ is primary-filter stable-vegetation EVI. $D_{j,t}$ equals 1 when response month t belongs to calendar month j and 0 otherwise, with j=4,…,10. The coefficient $\delta_{g,j}$ is the corresponding month-specific intercept. $\tau_{t}$ is the sequential monthly time index centered on the fitted sample and divided by 12 to express time in years. Its coefficient $\gamma_{g}$ is the linear EVI trend per year. The slope $\beta_{g,s,l}$ represents EVI change per 1-unit SPEI increase, and $\epsilon_{g,t}$ is the unexplained residual. Month indicators were fitted without an additional intercept. Dependence of the nuisance coefficients and residuals on the fitted combination is suppressed in Equation 6 for readability.

### 3.5 Partial correlation, significance, and best-combination determination

The Frisch–Waugh–Lovell procedure residualized EVI and each SPEI exposure against the growing-season month indicators and linear trend within the same pairwise sample. For a given combination (s,l), $\tilde{y_{g,t}}$ and $\tilde{x_{g,t}}$ denote the resulting EVI and SPEI residuals, respectively. Their combination indices are suppressed below. All sums are over matched valid response months t for that cell and combination. The partial Pearson correlation $r_{g,s,l}$ and estimated EVI slope $\beta_{g,s,l}$ were calculated using Equation 7:

(7) $$r_{g,s,l}=\frac{\sum_{t}\tilde{y_{g,t}}\tilde{x_{g,t}}}{\sqrt{\sum_{t}{\tilde{y_{g,t}}}^{2}\sum_{t}{\tilde{x_{g,t}}}^{2}}},\beta_{g,s,l}=\frac{\sum_{t}\tilde{y_{g,t}}\tilde{x_{g,t}}}{\sum_{t}{\tilde{x_{g,t}}}^{2}}$$

For each timescale–lag combination, the basin-wide summaries were weighted arithmetic means of the individual eligible-cell partial correlations and slopes. Each weight was the cell’s within-basin stable-vegetation area divided by the sum of that area over all eligible cells. The same 190 eligible cells entered all 52 primary summaries, regardless of significance. **Figure 3** summarizes cell-level estimates rather than regressions of pooled grid-month observations or basin-average time series. We averaged the cell-level correlations directly, without applying Fisher’s z transformation.

**Figure 3:**
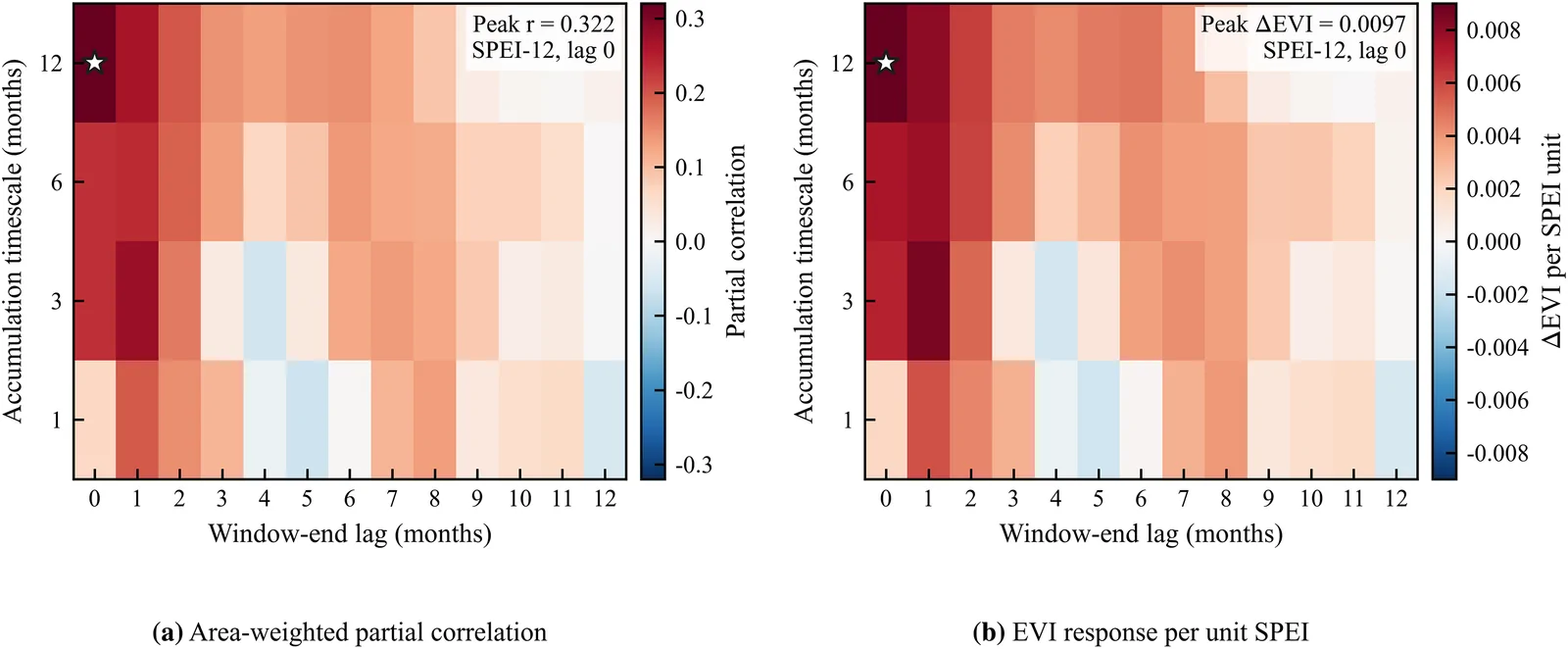
Basin-wide growing-season EVI responses across accumulation timescales and window-end lags. **(a)** Stable-vegetation-area-weighted arithmetic mean of cell-level partial correlations. **(b)** Corresponding weighted mean of cell-level EVI slopes per one-unit SPEI increase. Both panels include all 190 inference-eligible cells, not only detectable cells. Cell-level correlations are averaged directly, without applying Fisher’s z transformation. Stars mark the descriptive maximum among the 52 combinations.

Spatial eligibility was prespecified before inspecting response magnitudes. Cells required a basin fraction of at least 1%, native MODIS support of at least 90%, and an estimable stable-vegetation trend model. Inference additionally required at least 100 valid observations spanning at least 15 years and at least 10 observations for every April–October month. The directional hypothesis was positive moisture–greenness coupling: lower SPEI was associated with lower EVI after adjustment for month effects and trend. A one-sided test therefore targeted the largest signed partial correlation across all 52 combinations. Negative correlations remained in descriptive surfaces but were not tested as drought-suppression signals or converted to absolute values. The cell-wise maximum statistic $T_{g}$, truncated below at zero, was defined by Equation 8:

(8) $$T_{g}=\text{max}\{0,r_{g,s,l}\},s\in \{1,3,6,12\},l\in \{0,\ldots ,12\}$$

Calendar years were the temporal sampling units. Following the moving-block resampling principle for dependent observations (Künsch, 1989), we generated 1,999 synchronized 2-year null resamples of EVI control-variable residuals. SPEI remained at its true calendar position. Each replicate sampled 11 overlapping 2-year blocks with replacement from the 22-year record. Month order was retained within years, and the same year sequence was applied across cells and combinations. The 2-year blocks preserved within-year dependence and some adjacent-year dependence; 1- and 3-year blocks assessed sensitivity to this working choice. For replicate b, $T_{g}^{\left(b\right)}$ is the maximum statistic recalculated under the resampled null, and B is the number of null replicates. The one-sided empirical probability $p_{g}$ was calculated using Equation 9:

(9) $$p_{g}=\frac{1+\sum_{b=1}^{B}I(T_{g}^{\left(b\right)}\geq T_{g})}{B+1},B=1999$$

One omnibus $p_{g}$ per eligible cell was adjusted using the Benjamini–Hochberg false discovery rate procedure (Benjamini and Hochberg, 1995). Let $q_{g}$ denote the resulting adjusted p-value, distinct from the pixel-quality indicator $q_{i,k}$. An inferential best combination was reported only when the maximum partial correlation was positive and $q_{g}\leq \;0.05$. Exact ties favored the shorter lag and then the shorter accumulation timescale. A separate 1,999-replicate synchronized 2-year paired-block bootstrap resampled EVI and SPEI together and repeated selection among the 52 combinations. Selection stability was the proportion of replicates selecting the observed best timescale–lag combination, summarized across detectable cells. It quantifies selection uncertainty rather than the omnibus evidence of a positive association. Partial Spearman correlation and SPEI winsorization to [−5,5] provided additional sensitivity tests.

### 3.6 Vegetation type, environmental gradients, and alternative-response tests

Each 0.25°cell was assigned exclusively to the stable IGBP class occupying the largest stable-vegetation area. For vegetation-type response surfaces, dominant-class purity had to be at least 70%, with 80% assessed in sensitivity analysis. IGBP classes 1–5 were combined as forest, class 10 represented grassland, and class 12 represented cropland. Group response surfaces were weighted by stable-vegetation area and included all eligible cells in each group rather than only significant cells. Event composites used dominant-class labels for context without this additional purity restriction, as specified in Section 3.7; their type-specific populations therefore differ from those in **Figure 4**.

**Figure 4:**
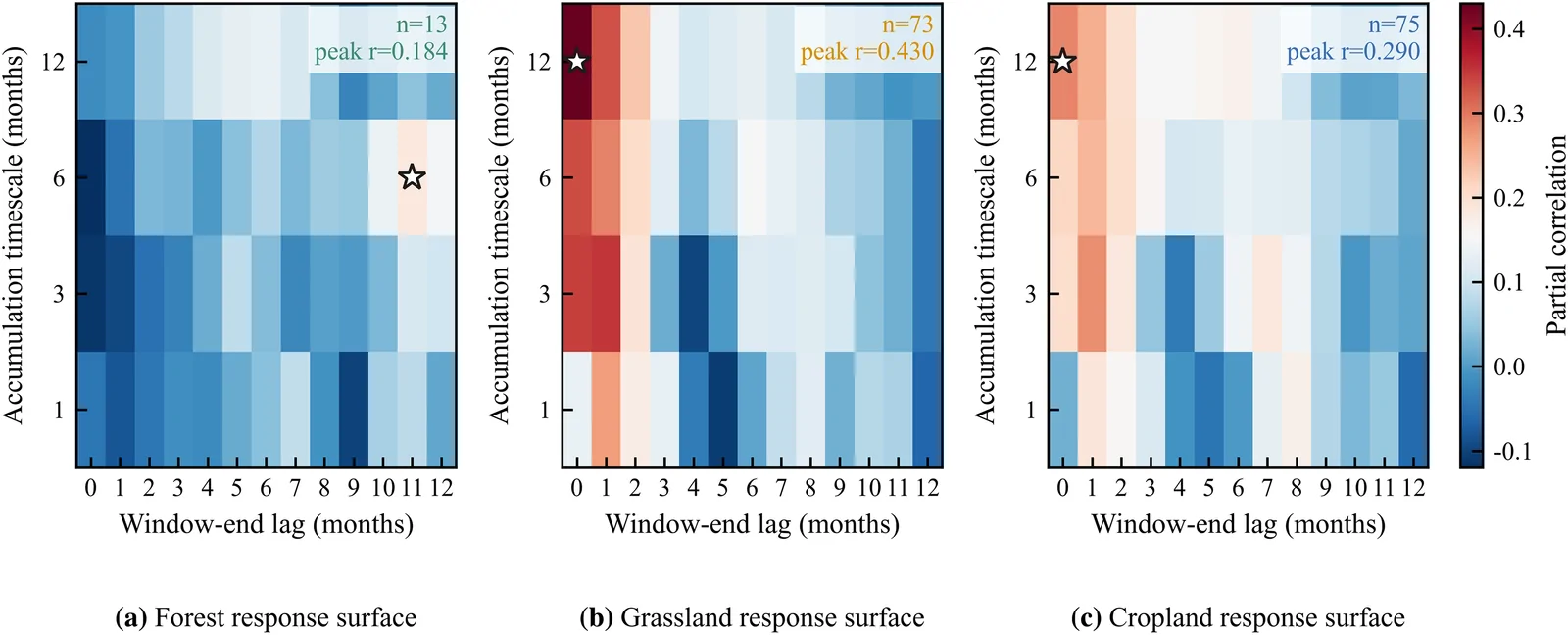
Drought response surfaces by dominant stable vegetation type: **(a)** forest, **(b)** grassland, and **(c)** cropland. Cells were assigned exclusively to the dominant stable IGBP group at a purity of at least 70%. Forest, grassland, and cropland contained 13, 73, and 75 eligible grid cells, respectively. All panels use the same color scale; stars mark the descriptive maximum among 52 combinations.

Environmental heterogeneity was evaluated using growing-season mean root-zone soil moisture, growing-season mean VPD, static elevation, and equipped-irrigation fraction. In Equation 10, $\theta_{1}$, $\theta_{2}$, and $\theta_{3}$ are ERA5-Land volumetric water contents for the 0–7, 7–28, and 28–100 cm layers. Each is expressed in $m^{3}m^{-3}$. The coefficients 0.07, 0.21, and 0.72 are the fractions of the 100 cm profile occupied by those layers. The thickness-weighted diagnostic $\theta_{0-100}$ has the same units:

(10) $$\theta_{0-100}=0.07\theta_{1}+0.21\theta_{2}+0.72\theta_{3}$$

Continuous variables were divided into low, medium, and high weighted tertiles. Complete 52-combination response surfaces were compared among strata, and descriptive associations between environmental conditions and response strength were calculated at the basin-wide peak combination, SPEI-12 at lag 0. Because soil moisture, VPD, and irrigation can share drivers with SPEI or lie on mediating pathways, these comparisons were not interpreted causally. Strict-filter EVI, EVI without the stable-land-cover filter, LAI, and GPP were re-estimated under the same temporal definition, controls, spatial support, and eligibility rules. We quantified response-surface similarity using the Pearson correlation between corresponding area-weighted partial correlations across the 52 timescale–lag combinations. Only combinations with finite values in both compared surfaces contributed to this calculation.

### 3.7 Drought-event composites and meteorological drought termination

SPEI-3 defined seasonal meteorological drought episodes, whereas the 52-combination analysis identified the window most closely associated with growing-season EVI. At least 2 consecutive months with SPEI-3 < −1 defined a candidate event. Qualifying drought segments separated by no more than 1 month were merged. Events were moderate when the minimum SPEI-3 was greater than −1.5 but below −1, severe when greater than −2 but at most −1.5, and extreme when at most −2. Left-censored events were excluded. Retained events had to intersect April–October in a primary-eligible cell. The same rules were applied separately to SPEI-1, SPEI-6, and SPEI-12 to assess sensitivity to event definition.

Using event onset as month 0, we extracted EVI residuals after calendar-month fixed effects and a linear trend from 3 months before through 12 months after onset. At each offset, basin-wide, severity-specific, and vegetation-specific means were weighted by stable-vegetation area over available observations. Type composites retained all events in cells labeled by their dominant stable IGBP class, without the 70% purity filter. They therefore describe cell-level vegetation context rather than pure-cover responses. Meteorological drought termination was confirmed in the second consecutive post-event month with SPEI-3 ≥ −0.5. This criterion indicates sustained exit from a specified threshold, not a return to mean climate or vegetation recovery. For event e, $t_{e,last}$ is the final month with SPEI-3 < −1, and $t_{e,confirm}$ is the termination-confirmation month. The confirmation interval $R_{e}$, measured in months, was defined by Equation 11:

(11) $$R_{e}=t_{e,confirm}-t_{e,last}$$

Events followed by another drought before confirmation were censored at the next event; those without confirmation before the record ended were right censored. $R_{e}$ runs from the last dry month to the second qualifying month. Its minimum is 2 months by construction. Censored events were excluded from these time summaries.

Uncertainty in event composites was estimated with 999 synchronized moving-block resamples of onset calendar years. For each replicate, 11 2-year blocks were sampled with replacement from the 22-year calendar frame. For each sampled onset year, we included the complete trajectories of all corresponding events. Events were repeated when their onset year was sampled more than once. The same year sequence was used for all cells and groups. This retained cross-cell co-occurrence within onset years, adjacent-year variability within blocks, and dependence among offsets of the same event. The 2.5th and 97.5th percentiles at each offset formed pointwise 95% intervals conditional on the observed event set. The 1- and 3-year blocks assessed block-length sensitivity. These pointwise intervals summarize trajectory uncertainty; no tests of between-group differences were performed.

### 3.8 Temporal summaries and processing-consistency checks

Descriptive temporal summaries used the fixed set of 190 primary inference-eligible cells. Each monthly EVI and SPEI-3 mean used the same cells with both variables available and normalized stable-vegetation-area weights. Monthly support could vary, but it was shared by the two variables. Annual values were means of the April–October monthly basin summaries. Ordinary least-squares regression of annual EVI on year separated its linear trend from annual residuals. This descriptive detrending differs from the grid-cell month-effect and trend controls used for response inference. Monthly climatology and interannual standard deviation used available EVI without imputation. Processing-consistency checks recomposited the 12 months of 2003 from source MODIS EVI and quality layers. We also compared gridded EVI with the monthly composites used to generate it. All 52 exposure alignments were checked against the source SPEI indices.

## 4 Results

### 4.1 Temporal variation in vegetation growth and drought

Across the 190 inference-eligible cells, mean growing-season EVI increased from 0.2454 in 2003 to 0.3363 in 2024. The fitted trend was 0.00414 EVI units per year (**Figure 5a**). Corresponding growing-season SPEI-3 was highest in 2003 (1.0607) and lowest in 2008 (−1.0159). After annual detrending, the 2003 EVI residual was close to zero (−0.00053), rather than a pronounced negative anomaly (**Figure 5b**). Low raw greenness early in the record can therefore coexist with relatively wet conditions because the variables have different temporal baselines.

**Figure 5:**
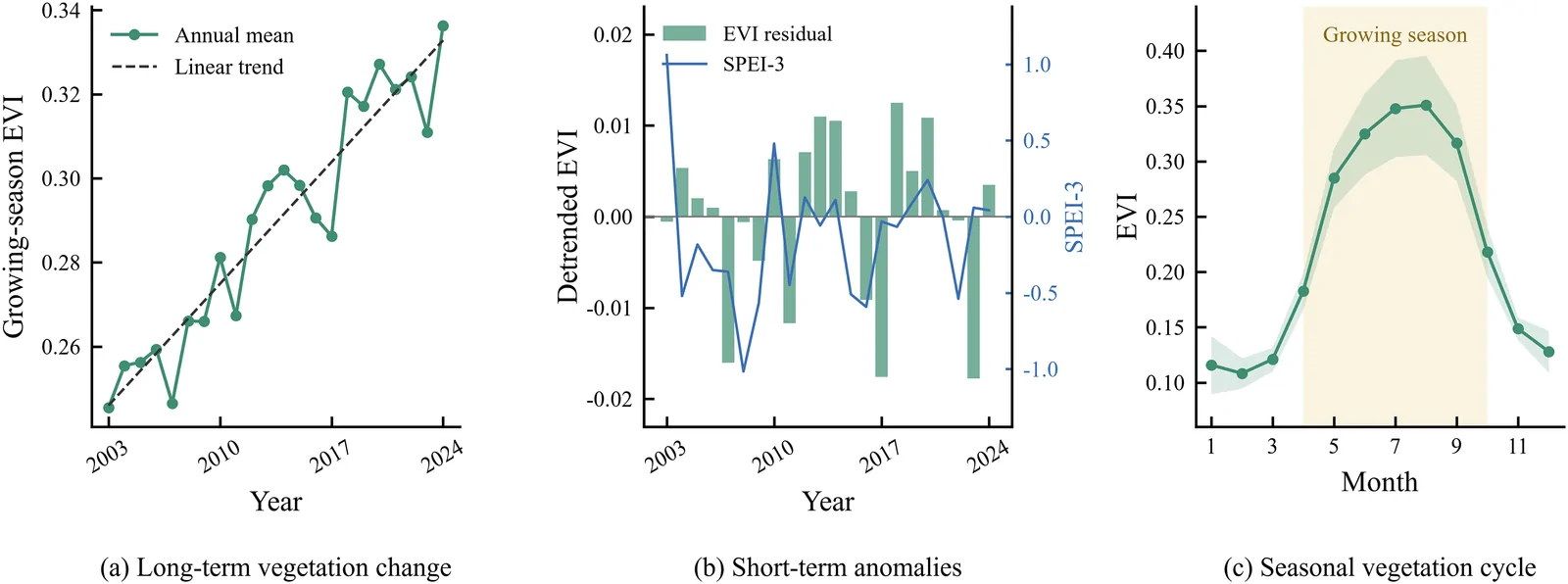
Descriptive temporal variation across the common support of 190 inference-eligible cells during 2003–2024. Each month, EVI and SPEI-3 use identical available cells and stable-vegetation-area weights. **(a)** April–October annual mean EVI and its ordinary least-squares linear trend, based on 22 annual summaries; the slope is 0.00414 EVI units per year. **(b)** Detrended annual EVI residuals (left axis) and April–October mean SPEI-3 (right axis). Residuals are in EVI units, whereas SPEI retains its source 1991–2020 standardization. **(c)** Monthly EVI climatology; shading denotes interannual standard deviation, not a confidence interval, and April–October is highlighted. Missing observations are not interpolated. The annual detrending differs from the grid-cell month-effect and trend controls used for response inference.

The mean seasonal EVI cycle was unimodal, rising from 0.1210 in March to 0.3478 in July and peaking in August at 0.3509 before declining. February had the lowest monthly mean (0.1083; **Figure 5c**). April–October encompassed the principal phases of canopy development, peak greenness, and senescence and was retained as the growing season.

### 4.2 Basin-wide accumulation-timescale and window-end-lag response

After adjustment for growing-season month effects and a linear trend, EVI–SPEI association strength varied across accumulation timescales and window-end lags (**Figure 3**). The basin-wide area-weighted partial correlation peaked at 0.3216 for SPEI-12 at lag 0, with a slope of 0.00971 EVI units per SPEI unit (**Figures 3a, b**). A 1-unit SPEI decline thus corresponded to a mean EVI decrease of 0.00971. Six- and 12-month windows showed relatively continuous positive associations. The 12-month response was strongest at lags 0–2 and weakened thereafter. 1- and 3-month responses were more variable, with weak negative associations at some intermediate or long lags. The correlation and raw-EVI slope surfaces were closely aligned.

Alternative data filters constrained but did not overturn this pattern. The strict EVI analysis retained 64 eligible cells and had a response-surface similarity of 0.9710 with the main result. Removing the stable-land-cover filter retained 194 cells and yielded a similarity of 0.9974; both sensitivity surfaces also peaked at SPEI-12, lag 0. The peak partial Spearman correlation was 0.3057, and the peak partial correlation after SPEI winsorization remained 0.3216. The GPP analysis retained only 20 eligible cells and peaked at SPEI-6, lag 0 (r = 0.2789). No LAI cell simultaneously satisfied the prespecified coverage and sample-size thresholds, so LAI was not used for equivalent inference.

Relaxing spatial valid-area coverage from 0.70 to 0.50 increased eligibility to 228 cells, of which 143 were detectable, representing 66.64% of that scenario’s eligible stable-vegetation area (**Table 2**). The basin-wide peak remained SPEI-12, lag 0, with mean partial r = 0.3041 and slope = 0.00936. Thus, the peak combination persisted when more cells were included in the analysis. Its response magnitude changed, as did the eligible vegetation area used to calculate the percentage with a detected association.

**Table 2:** Sensitivity to spatial valid-area coverage and temporal block length.

Setting	Area coverage	Block length (years)	Eligible cells	Detected cells/area (%)	Peak mean partial r
Primary	0.70	2	190	126/69.68	0.3216
Relaxed coverage	0.50	2	228	143/66.64	0.3041
1-year blocks	0.70	1	190	120/65.92	0.3216
3-year blocks	0.70	3	190	119/65.98	0.3216

All settings peak descriptively at SPEI-12, lag 0. Detected area is the percentage of eligible stable-vegetation area within that setting; relaxing coverage changes this denominator. Block length changes resampling-based inference, not the estimated descriptive surface. Each null test uses 1,999 replicates with BH-FDR q≤ 0.05.

### 4.3 Spatial heterogeneity in scale–lag responses

Scale–lag responses varied markedly across the basin (**Figure 6**). Of the 190 cells meeting prespecified inference criteria, 126 passed the cell-wise maximum-statistic test and BH-FDR correction. These cells represented 66.32% of eligible cells and 69.68% of eligible stable-vegetation area. The 64 eligible cells without a corrected positive association were concentrated mainly in parts of the south-central and eastern basin. Stronger positive associations formed more continuous patterns in the western and north-central basin and in several eastern localities (**Figure 6c**).

**Figure 6:**
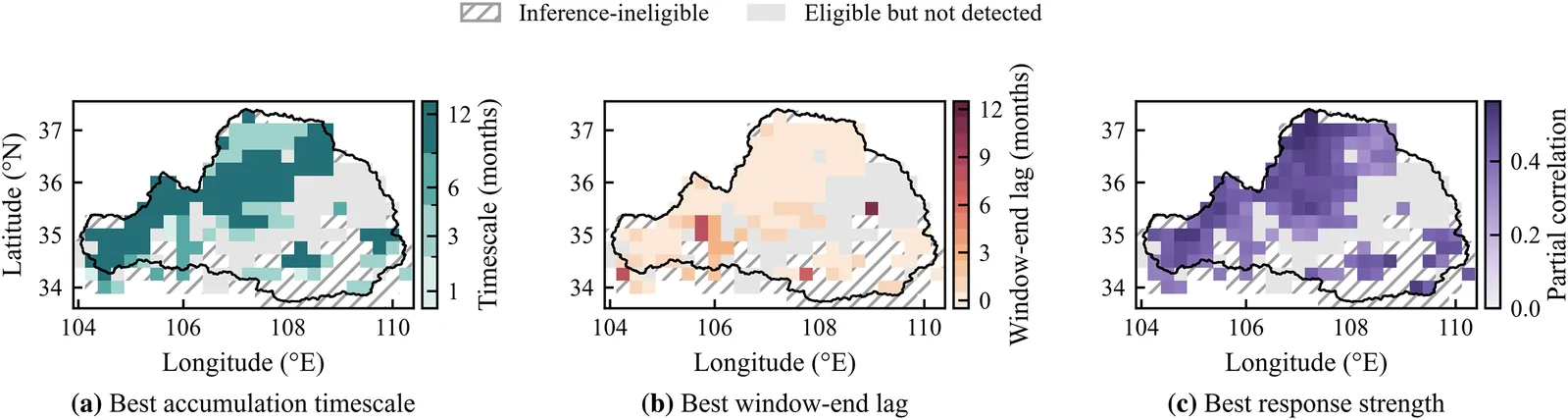
Spatial patterns of accumulation-timescale and window-end-lag responses. **(a)** Best accumulation timescale, **(b)** best window-end lag, and **(c)** corresponding partial correlation for cells passing the cell-wise maximum-statistic test and BH-FDR correction. Hatching denotes inference-ineligible cells, light gray denotes eligible cells without a detected positive association, and white denotes areas outside the basin. The maps show the selected timescales, lags, and corresponding partial correlations. Selection is uncertain, with a median paired-bootstrap reselection frequency of 53.1% across detectable cells.

Among the 126 detectable cells, 76 selected a 12-month accumulation period (60.32%), 36 selected 3 months (28.57%), and only 3.17% and 7.94% selected 1 and 6 months, respectively (**Figure 6a**). Best window-end lags were even more concentrated: 91 cells selected lag 0 (72.22%), 21 selected lag 1 (16.67%), and all remaining lags together accounted for 11.11% (**Figure 6b**). Long accumulation windows ending near the response month therefore predominated, although short-timescale or longer-lag behavior persisted locally.

The specific timescale–lag combination selected for each cell was less stable than the detection of a positive association. Across the 126 detectable cells, the paired-bootstrap reselection frequency of the observed best combination had a median of 53.1% (interquartile range, 38.3–63.6%). In 110 cells, this combination was also the most frequently selected across bootstrap replicates. With 1-, 2-, and 3-year null blocks, 120, 126, and 119 cells were detected, covering 65.92%, 69.68%, and 65.98% of the same eligible stable-vegetation area, respectively (**Table 2**).

### 4.4 Vegetation-type and environmental differences

Dominant stable vegetation types exhibited distinct response surfaces (**Figure 4**). Grassland showed the strongest response, peaking at SPEI-12, lag 0 (r = 0.4299), and maintained strong positive associations for the 12-month index at lags 0–2 (**Figure 4b**). Cropland also peaked at SPEI-12, lag 0 (r = 0.2899), with association strength generally declining as the window-end lag increased (**Figure 4c**). Forest responses were weaker and had a descriptive peak at SPEI-6, lag 11 (r = 0.1844; **Figure 4a**). Forest, grassland, and cropland contained 13, 73, and 75 eligible cells, respectively.

At 80% dominant-class purity, eligible forest, grassland, and cropland sample sizes decreased to 9, 70, and 63 cells. Their peak combinations remained SPEI-6 at lag 11, SPEI-12 at lag 0, and SPEI-12 at lag 0, with mean partial correlations of 0.1880, 0.4296, and 0.2901, respectively. The relative ordering of response strengths persisted at the higher purity threshold.

Elevation showed the clearest environmental gradient (**Table 3**). Peak partial correlations increased from 0.2758 at low elevation to 0.3131 at medium elevation and 0.3855 at high elevation. Medium- and high-elevation strata peaked at SPEI-12, lag 0, whereas the low-elevation stratum peaked at SPEI-3, lag 1. At the fixed SPEI-12, lag 0 combination, response strength had a Spearman correlation of 0.3308 with elevation. Root-zone soil moisture, VPD, and equipped-irrigation strata showed no stable monotonic pattern, and absolute correlations at the fixed combination were ≤0.10.

**Table 3:** Peak EVI accumulation-timescale and window-end-lag responses across environmental gradients.

Environmental variable	Stratum	Grid cells	Peak accumulation (months)	Peak window-end lag (months)	Peak partial r
Root-zone soil moisture	Low	60	12	0	0.2986
Root-zone soil moisture	Medium	57	12	0	0.3314
Root-zone soil moisture	High	73	12	0	0.3349
VPD	Low	72	12	0	0.3178
VPD	Medium	55	12	0	0.3000
VPD	High	63	12	0	0.3464
Elevation	Low	61	3	1	0.2758
Elevation	Medium	55	12	0	0.3131
Elevation	High	74	12	0	0.3855
Equipped-irrigation fraction	Low	71	12	0	0.3648
Equipped-irrigation fraction	Medium	60	12	0	0.2590
Equipped-irrigation fraction	High	59	12	0	0.3412

Each group peak was selected as the maximum area-weighted partial correlation among four accumulation timescales and 13 window-end lags.

### 4.5 Vegetation trajectories and meteorological drought termination

Across 2,252 SPEI-3 grid-cell drought events, the area-weighted EVI residual decreased from 0.00977 one month before onset to 0.00118 at onset and −0.00909 one month afterward. The pointwise 95% interval at month 1 was −0.01448 to −0.00439 using synchronized 2-year onset-year block resampling. The 1- and 3-year blocks also gave negative month-1 intervals (−0.01641 to −0.00330 and −0.01539 to −0.00464, respectively). Extreme events reached their minimum residual 1 month after onset (−0.01622), whereas moderate events reached a smaller minimum after 2 months (−0.00704); severe-event mean anomalies remained negative over a longer subsequent interval (**Figure 7a**).

**Figure 7:**
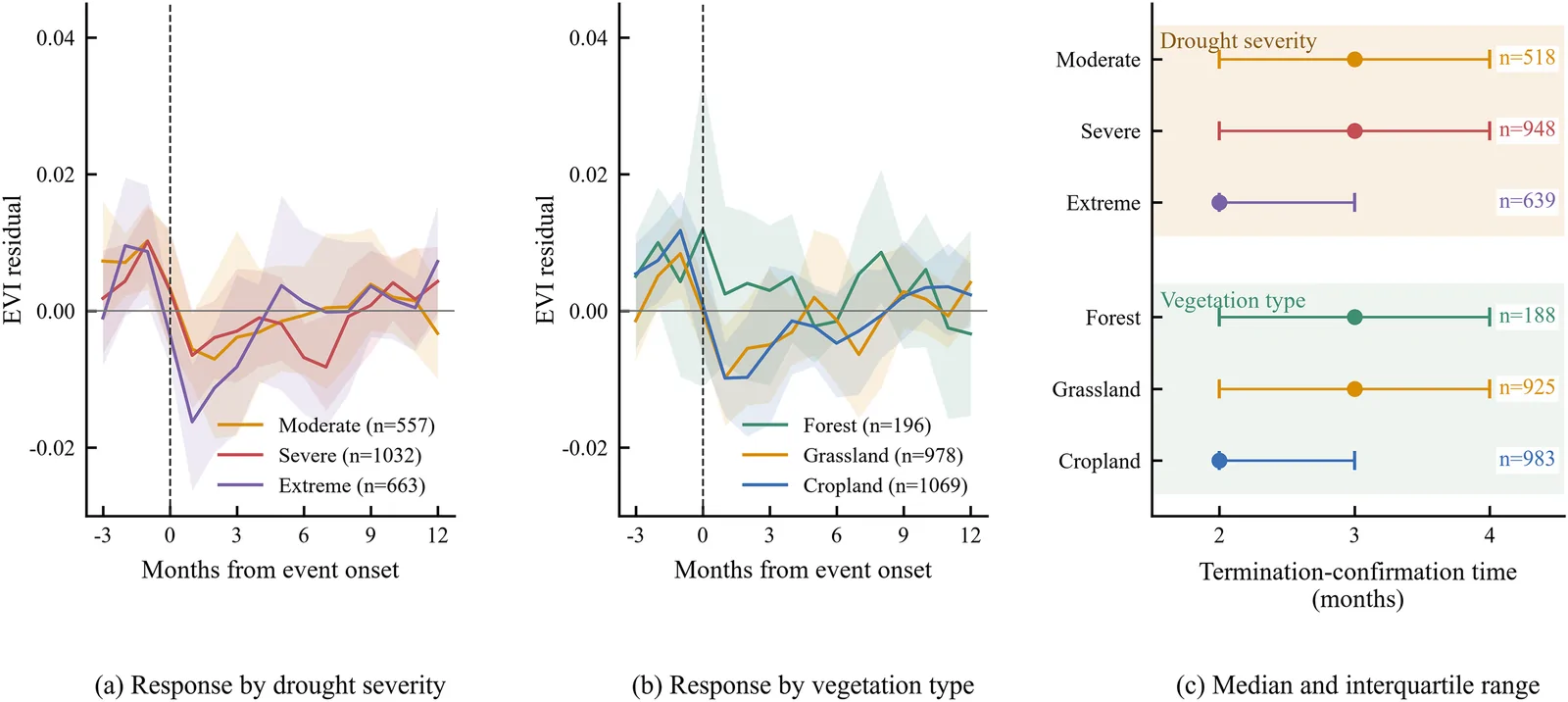
Vegetation trajectories and meteorological drought termination confirmation. **(a, b)** Lines show stable-vegetation-area-weighted EVI residuals; bands are pointwise 95% percentile intervals from 999 synchronized 2-year moving-block resamples of event onset years. Whole event trajectories and all cells sharing sampled years are retained together. n denotes grid-cell events, not independent regional droughts; the dashed line marks onset. The total is 2,252 events; the three vegetation groups sum to 2,243, and nine savanna-dominated events are included only in the overall total. Type labels are contextual and do not impose the 70% purity filter used in **Figure 4**. **(c)** For uncensored events, points show median time from the last month below −1 to the second consecutive month with SPEI-3 ≥ −0.5; bars show the interquartile range, not a confidence interval. n denotes events with confirmed termination. The overall confirmed total is 2,105, including nine other-vegetation events not displayed by type.

The event sample comprised 196 forest, 978 grassland, 1,069 cropland, and nine other-vegetation events. The nine events occurred in cells dominated by IGBP class 9 (savannas); they were included in the total but not in the three displayed type groups. Grassland and cropland mean residuals reached −0.00977 and −0.00982 one month after onset, respectively. Forest mean trajectories varied less, but their intervals included substantial uncertainty (**Figure 7b**). Among 2,105 events with uncensored termination confirmation, the median confirmation time was 3 months (interquartile range, 2–3 months; **Figure 7c**). Medians were 3, 3, and 2 months for moderate, severe, and extreme events, and 3, 3, and 2 months for forest, grassland, and cropland.

Event definition changed the episode population and response timing. Applying identical rules to SPEI-1, SPEI-6, and SPEI-12 identified 1,059, 1,837, and 1,200 events, respectively. Their overall mean EVI minima within months 0–12 occurred at months 2, 2, and 0, with residuals of −0.00794, −0.01283, and −0.01254. Median termination-confirmation times among their uncensored events were 4, 3, and 3 months. The timing of the EVI minimum therefore depended on the SPEI timescale used to define drought events.

## 5 Discussion

### 5.1 Ecological meaning of the dominant long accumulation window

The basin-wide peak at SPEI-12 and lag 0 indicates an association with the 12-month water balance ending in the observation month. SPEI is explicitly multiscalar, with short windows representing transient anomalies and longer windows integrating cross-seasonal deficits (Vicente-Serrano et al., 2010). In this context, SPEI-12 summarizes the preceding year’s water balance in relation to current vegetation state.

The long-window signal agrees with evidence of cumulative water limitation, although exact optima depend on the study design and region. Dominant SPEI timescales vary among climatic regions and vegetation types across China (Zhang et al., 2017). On the Loess Plateau, arid-region productivity more often responds to SPEI-3, whereas semi-arid and semi-humid areas commonly respond to SPEI-12 or SPEI-24 (Zhao et al., 2018). Grassland analyses have reported 5–10-month accumulation periods and 2–3-month lags (Zhao et al., 2020). Yellow River Basin studies also find stronger cumulative than lagged effects (Zhan et al., 2022). National analyses explicitly document both dimensions, reinforcing the need to compare their definitions rather than assume one universal optimum (Wang et al., 2023a). In a semi-arid steppe, Liu et al. (2024) found that precipitation context altered lagged and cumulative controls on the end of the growing season. Their phenological endpoint captures a different aspect of vegetation timing from monthly EVI. Response variables, seasonal context, spatial support, detrending, and exposure-window definitions can all contribute to differences among studies.

A 12-month signal is consistent with storage of antecedent precipitation in soil and groundwater and with vegetation access to water of different ages. Water-limited ecosystems can shift toward deeper soil, rock, or groundwater sources during drought (Miguez-Macho and Fan, 2021). This interpretation is consistent with hydrological memory, although rooting depth and groundwater uptake were not directly observed. Operational monitoring can use SPEI-12 to screen cross-seasonal stress while retaining SPEI-3 for localized short-term deterioration.

### 5.2 Vegetation and environmental heterogeneity

Vegetation-type contrasts show why a single basin-wide parameter is insufficient. Grassland and cropland both peaked at SPEI-12, lag 0, with a stronger grassland association. Forest had a weaker descriptive maximum at SPEI-6, lag 11. Stronger grassland and cropland responses are consistent with national and Yellow River Basin evidence (Zhang et al., 2017; Zhan et al., 2022). Grassland sensitivity exceeded forest sensitivity in a recent Tibetan Plateau study of compound drought–heat stress, although its stress index and seasonal design differed from ours (Li et al., 2026). Rainfall-manipulation experiments also show cumulative productivity losses in Eurasian grasslands, but substantial continental differences caution against a universal grassland response (Yu et al., 2025). Rooting depth, canopy adjustment, phenology, and irrigation are plausible contributors to type contrasts; access to older water can buffer vegetation against short deficits (Miguez-Macho and Fan, 2021). The forest peak remains uncertain because only 13 cells met the type-purity and inference criteria.

The elevation gradient was the clearest environmental contrast. Stronger high-elevation associations may reflect covariation among temperature limitation, soil depth, topographic convergence, growing-season length, and vegetation composition rather than a direct elevation effect. Upper-basin highlands and lowland agricultural zones also differ in evaporative demand and managed water supply, so the same SPEI anomaly need not impose the same vegetation stress.

Weak monotonic differences across soil-moisture, VPD, and irrigation strata do not imply that those factors are unimportant. Atmospheric demand and soil water constrain ecosystem water and carbon exchange through different pathways, and warming can amplify VPD limitation (Novick et al., 2016). However, VPD and soil moisture correlate with SPEI and may lie on mediating pathways, while the static irrigation layer cannot capture monthly water delivery.

### 5.3 Vegetation loss and meteorological drought termination

The scale–lag surface summarizes associations across growing seasons, whereas event composites describe trajectories around seasonal SPEI-3 onset. The negative month-1 composite indicates early deterioration under this event definition, despite a basin-wide scale–lag peak at lag 0. Window-end lag is measured from the end of an accumulation window; event offset starts at the first month of a qualifying episode. The SPEI-1, SPEI-6, and SPEI-12 diagnostics placed mean post-onset EVI minima within months 0–2, so they do not support a universal 1-month delay. Seasonal divergence in Tibetan Plateau responses to compound drought–heat stress further illustrates the importance of timing and stress definition (Li et al., 2026).

The median SPEI-3 termination-confirmation time was 3 months among uncensored events. The endpoint is the second consecutive month at or above −0.5, so the interval includes a confirmation requirement rather than a return to mean climate. The 2-month median for extreme events is the definitional lower bound and does not demonstrate faster ecological recovery. Greenness, productivity, terrestrial water storage, and tree growth require their own recovery endpoints. Drought effects can persist across seasons or years after meteorological conditions improve (Anderegg et al., 2015; Schwalm et al., 2017; Wu et al., 2024). Using multiple satellite indicators, Li et al. (2023) found that spring phenology influenced ecosystem recovery and that many ecosystems did not recover within 1 growing season. Their ecological recovery endpoints differ from the meteorological threshold used here.

### 5.4 Implications for restoration and drought management

The SPEI-12 peak supports screening established vegetation with accumulated water conditions rather than current-month precipitation alone. Revegetation has increased water use across the Loess Plateau, where some locations may be approaching water-resource limits (Feng et al., 2016). Recent evidence also shows that Loess Plateau greening can coexist with declining resilience (Wang et al., 2023b). Rising EVI should therefore not be interpreted as proof of reduced drought vulnerability. Restoration decisions require additional observations of newly converted vegetation, water demand, and management.

Monitoring priorities should differ among vegetation types and environmental settings. Grassland and high-elevation stable-vegetation zones warrant combined tracking of SPEI-12, post-onset EVI change, and meteorological drought termination confirmation. Low-elevation areas should retain SPEI-3 and short-lag indicators. Cropland interpretation requires actual irrigation, crop-calendar, and soil-moisture data, whereas forest thresholds require stand, tree-ring, or productivity validation. Vegetation monitoring should continue after meteorological drought termination, especially after extreme events or closely spaced droughts. These recommendations support basin-scale drought-risk screening with 0.25° data; local management requires finer-resolution observations and site-specific assessment.

### 5.5 Robustness, limitations, and future directions

Strict EVI filtering, relaxation of valid-area coverage, removal of the stable-land-cover filter, partial Spearman correlation, and SPEI winsorization supported a consistent descriptive basin-wide SPEI-12, lag 0 peak. Increasing type purity retained the three type-specific peak combinations, although the forest sample contracted to nine cells. The number of detected cells varied with block length, ranging from 119 to 126 across 1–3-year blocks. Across detected cells, the median bootstrap reselection frequency of the observed best timescale–lag combination was 53.1%. These results support the broad response pattern more strongly than the precise lag selected for each cell. Synchronized resampling preserves temporal variation shared across cells in the analysis. Maximum-statistic testing accounts for selection among candidate combinations, and BH-FDR addresses multiple comparisons across cells.

Several limitations constrain inference. Within persistent land-cover classes, degradation, succession, and management changes may still occur. The stability filter excludes some conversion trajectories, so the findings concern persistent vegetation rather than the effects of land conversion. The 0.25° grid also cannot resolve slope aspect, soil depth, or field-scale irrigation. No independent field measurements were used to assess local product accuracy or validate the inferred vegetation responses. EVI measures canopy greenness, while no LAI cell met equivalent eligibility criteria. Only 20 GPP cells qualified, supporting a limited sensitivity comparison. Event intervals reflect shared onset-year variability and short temporal dependence but remain conditional on the observed events and the 22-year calendar record. Finally, partial correlations and analyses of environmental strata cannot separate causal and mediating effects of water balance, VPD, soil moisture, and managed water supply.

Future work should combine meteorological stations, soil-moisture measurements, and groundwater observations to test the proposed role of antecedent water storage. Vegetation-type-specific EVI and GPP records, annual irrigation information, and crop calendars would help distinguish biological responses from management effects. Distributed-lag or event-matching designs could then assess concurrent accumulation, delayed responses, and drought legacies before basin-scale associations are translated into local management thresholds.

## 6 Conclusions

Using MODIS EVI and ERA5-Drought SPEI, we characterized vegetation–drought associations on the native 0.25° grid of the Weihe River Basin. The analysis distinguished drought accumulation timescales, window-end lags, and vegetation responses around drought onset. It covered persistent land-cover classes during 2003–2024, requiring at least 18 valid annual classifications and a modal-class fraction of at least 80%.

1. Basin-wide vegetation associations were dominated by long-term accumulated water conditions. The area-weighted response peaked at SPEI-12, lag 0 (r = 0.3216). A one-unit SPEI decrease corresponded to a mean EVI decrease of 0.00971. Of 190 eligible cells, 126 passed BH-FDR correction, covering 69.68% of eligible stable-vegetation area. Monitoring should include the 12-month water balance ending in the response month.
2. Response surfaces showed spatial and ecological heterogeneity. Grassland and high-elevation groups had stronger descriptive associations, supporting differentiated monitoring of established vegetation. However, the median bootstrap reselection frequency of the observed best timescale–lag combination was only 53.1% across detectable cells. Local scale–lag maps can therefore inform spatially targeted drought-risk screening.
3. Across 2,252 SPEI-3 drought events, the mean EVI residual reached −0.00909 one month after onset; its 2-year onset-year block-bootstrap interval remained negative. The timing of the overall minimum varied between months 0 and 2 under alternative SPEI event definitions. Among 2,105 uncensored SPEI-3 events, meteorological drought termination was confirmed a median of 3 months after the last dry month (interquartile range, 2–3 months). Practical monitoring should pair long-term accumulated water conditions with early event trajectories and continue vegetation observations after meteorological drought termination is confirmed.

## Data Availability

All primary datasets are publicly available from NASA LP DAAC (MOD13A1.061, MCD12Q1.061, MCD15A3H.061, and MOD17A2HGF.061), the Copernicus Climate Data Store (ERA5-Drought and ERA5-Land), the Copernicus DEM program, TerraClimate, and FAO AQUASTAT GMIA version 5; persistent identifiers are provided in the References and **Table 1**. SRTM elevation data are publicly available through the USGS EROS archive and EarthExplorer (https://earthexplorer.usgs.gov/). Derived data and analysis code supporting the reported findings are available from the corresponding author on reasonable request.
